# RaptGen-UI: an integrated platform for exploring and analyzing the sequence landscape of HT-SELEX experiments

**DOI:** 10.1093/bioadv/vbaf120

**Published:** 2025-05-23

**Authors:** Ryota Nakano, Natsuki Iwano, Akiko Ichinose, Michiaki Hamada

**Affiliations:** Graduate School of Advanced Science and Engineering, Waseda University, Tokyo, 169-8555, Japan; Graduate School of Advanced Science and Engineering, Waseda University, Tokyo, 169-8555, Japan; Graduate School of Advanced Science and Engineering, Waseda University, Tokyo, 169-8555, Japan; Graduate School of Advanced Science and Engineering, Waseda University, Tokyo, 169-8555, Japan; Cellular and Molecular Biotechnology Research Institute (CMB), National Institute of Advanced Industrial Science and Technology (AIST), Tokyo, 135-0064, Japan; Graduate School of Medicine, Nippon Medical School, Tokyo, 113-8602, Japan

## Abstract

**Summary:**

RaptGen-UI provides intuitive graphical user-interface of the system exploring and analyzing the sequence landscape of high-throughput (HT)-SELEX (Systematic Evolution of Ligands by EXponential enrichment) experiments through machine learning-driven visualization with optimization capabilities. This software enables wet-lab researchers to efficiently analyze HT-SELEX dataset and optimize RNA aptamers without requiring extensive computational expertise. The containerized architecture ensures secure local deployment and supports both of high-performance Graphics Processing Unit (GPU) acceleration and CPU-only environments, making it suitable for various research settings.

**Availability and implementation:**

This software is a web-based application running locally on the user’s PC. The frontend is constructed using Next.js and Plotly.js with TypeScript, while the backend is developed using FastAPI, Celery, PostgreSQL RDBMS, and Redis with Python. Each module is encapsulated within Docker containers and deployed via Docker Compose. The system supports both CUDA GPU and CPU-only environments. Source code and documentation are freely available at https://github.com/hmdlab/RaptGen-UI.

## 1 Introduction

Aptamers are single-stranded oligonucleotides that bind their target molecules with high affinity and specificity through structural and chemical interactions. They are analogous to antibodies but have the advantages of being smaller, broader in the target range, and easier to produce (Zhou and Rossi 2017). Aptamers, particularly RNA aptamers, are primarily employed for medical purposes, such as pharmaceuticals and drug delivery technologies. In addition, they are also increasingly utilized outside medical applications, such as biosensing, where they serve as environmental biomarkers (Wang *et al.* 2011). Therefore, the identification and optimization of RNA aptamers are crucial for various fields.

The identification of RNA aptamers is typically undertaken through utilization of the SELEX (Systematic Evolution of Ligands by EXponential enrichment) methodology (Ellington and Szostak 1990, Tuerk and Gold 1990). SELEX employs a systematic, iterative selection process of aptamers that encompasses selection, partition, and amplification steps. With the advent of high-throughput sequencers and the subsequent advancements in information engineering, the SELEX process has now been augmented to incorporate sequence-based analysis, thereby facilitating the identification of aptamer sequences with greater efficiency. A significant number of extant methods have been employed to select binding sequences based on data derived from RNA pools obtained from each round of SELEX experiments.

Despite the considerable advancements in computational methodologies for sequence identification from SELEX data, the availability of user-friendly graphical interfaces remains limited (Komarova *et al.* 2020). The development of cross-platform systems that wet-lab researchers can employ without extensive computational skills or bioinformatics expertise, and that function effectively across diverse computational environments is imperative for mitigating the discrepancy between computational techniques and practical laboratory applications, thereby fostering the broader adoption of HT-SELEX and aptamer technologies in the field. Among the limited number of available methods, AptaSUITE (Hoinka *et al.* 2018) is noteworthy for its provision of a wide array of tools through an intuitive graphical user interface (GUI). This product, developed in Java to ensure cross-platform compatibility, has been extensively adopted in aptamer research and related communities. Another notable example is APTANI2, which has been updated from its previous version to incorporate a user-friendly interface for non-expert users (Caroli *et al.* 2020). These developments underscore the growing importance of implementing GUIs in the realm of aptamer research.

The proposed software, RaptGen-UI, is designed to re-implement and streamline the identification and optimization of RNA aptamers using RaptGen (Iwano *et al.* 2022), a generative model that employs Latent Space Bayesian Optimization (LSBO) techniques. LSBO is a powerful optimization method that uses latent variable models like Variational Autoencoders (VAEs) to build surrogate functions in the latent space. This technique enables iterative optimization in continuous space to maximize desired properties. While LSBO was initially proposed by Gómez-Bombarelli *et al.* (2018), RaptGen further refined this concept by incorporating profile Hidden Markov Models (pHMMs) (Eddy 1998) into the VAE decoder. This approach accommodates insertions and deletions, improving motif acquisition. Subsequent optimization is performed with assays such as Surface Plasmon Resonance (SPR) (Piliarik *et al.* 2009), enabling the identification of high-affinity sequences. Generative models offer an advantage in that they can propose sequences not observed in the original dataset. RaptGen-UI provides an intuitive interface for wet-lab researchers to interact with and operate on the cutting-edge generative model, enabling them to identify and optimize RNA aptamers efficiently.

## 2 Software design and implementation

RaptGen-UI is a software designed to minimize manual workload while enabling the identification and iterative optimization of RNA aptamer candidates. In addition, our software has been engineered with a system design that carefully considers the anticipated use cases and the following specific environmental factors prevalent in aptamer research.

**Containerized:** Given the time-intensive nature of machine learning processes, the tool employs containerized infrastructure to execute tasks in the background. This containerization also ensures compatibility across various environments, eliminates the need for users to install or configure dependencies, and simplifies the deployment process. As the actual implementation, all containers are orchestrated using Docker Compose, enabling straightforward deployment with a single docker compose up command, regardless of the user’s environment.

**GPU acceleration:** The tool provides a version that leverages Graphics Processing Unit (GPU) acceleration as well as a standard version intended for environments not equipped with GPU capabilities. This enables the tool to accommodate computationally intensive tasks. The GPU-accelerated version utilizes CUDA for faster VAE training, while the standard version is designed to run on CPUs.

**Data confidentiality:** When sequences are identified as having the potential to be intellectual property, data confidentiality must be considered. To address this concern, the software operates entirely in a local environment, ensuring secure and rapid sequence identification without requiring external data transfer. Additionally, web technologies enable server-client separation, thereby allowing users to interact with results on local personal computers (PCs) while offloading machine learning tasks to GPU servers. Users can access the remote server via Secure Shell (SSH) tunneling and port forwarding, ensuring secure data transmission.

RaptGen-UI is built with Next.js and TypeScript for the frontend and FastAPI with Python for the backend API server. Data storage utilizes PostgreSQL with SQLalchemy. Celery with Python is used to distribute task queues for high-cost functions such as training VAE models. Redis is used as a message broker for Celery. All components are containerized for compatibility across environments and to eliminate dependencies apart from GPU availability. Docker Compose orchestrates these containers, allowing users to initiate the system with a single command. A mono-repo design centralizes project files, allowing users to access updates and provide feedback through GitHub.

## 3 Results

**Figure 1.**
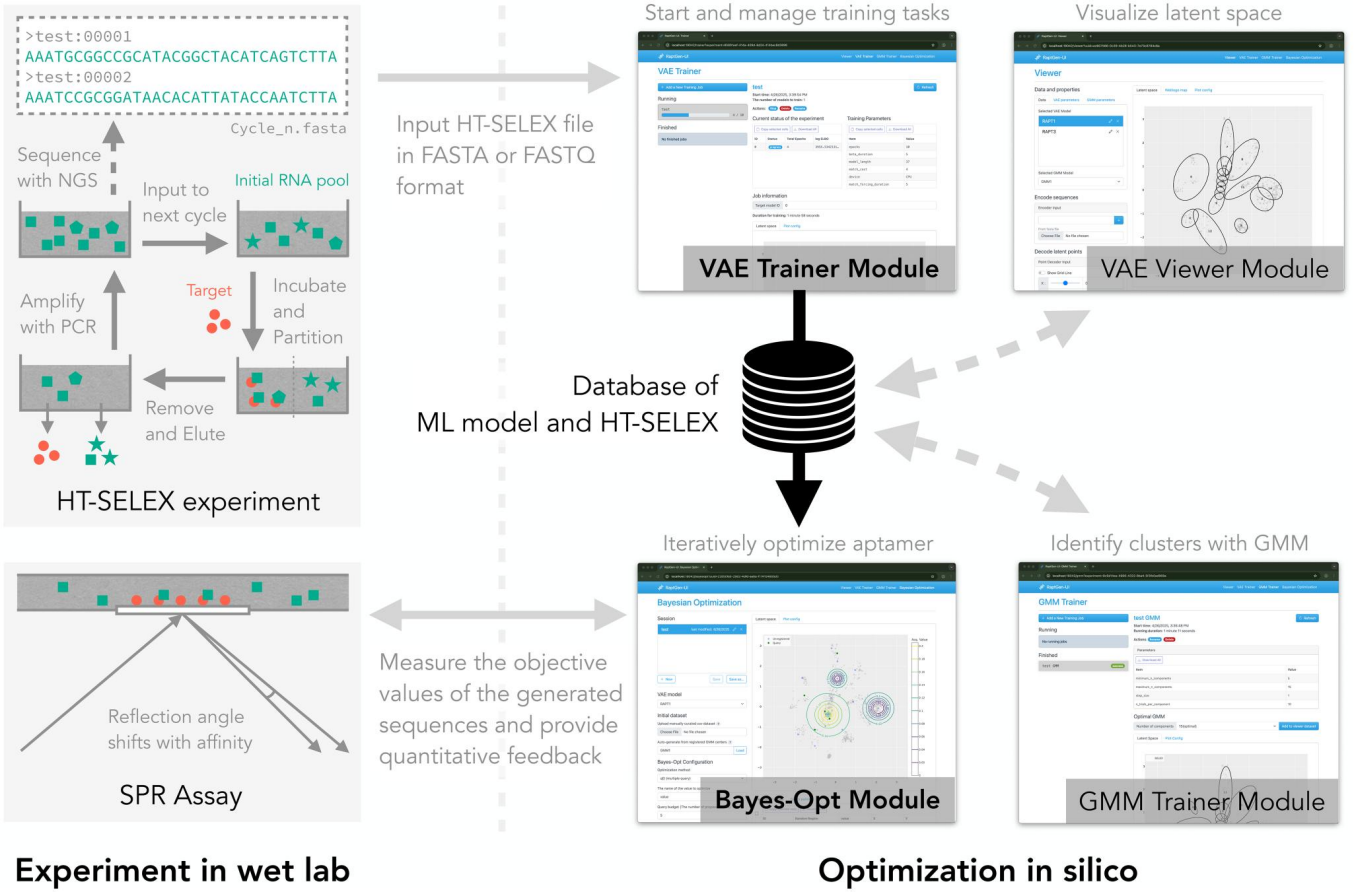
Overview of RaptGen-UI and its workflow. Users perform HT-SELEX experiments and upload the resulting FASTA or FASTQ files to the VAE Trainer. In the VAE Trainer module, users configure the parameters for model training and initiate the training process. The training duration depends on the size of the dataset and parameters, typically ranging from several hours to a few days. Once the model training is complete, users select a model for downstream analyses and save it to the database. The saved models can be interactively analyzed in the VAE Viewer. In the VAE Viewer, users can map sequences to the latent space, decode sequences from the latent space, and visualize motifs using weblogo. Furthermore, the GMM Trainer module facilitates cluster identification by training GMMs on the latent space for the models stored in the database. The centroids of the obtained clusters can be utilized as initial points for Bayesian optimization. The Bayes-Opt module performs Bayesian optimization over the latent space of the models stored in the database. Users receive coordinates and sequences proposed by the Bayes-Opt module and perform binding affinity assays, such as SPR assays, on the suggested sequences. The obtained results serve as oracles for the objective function in Bayesian optimization, enabling the exploration of sequences with high binding affinity.

RaptGen-UI is composed of four primary components: the VAE Trainer, the VAE Viewer, the GMM (Gaussian mixture model) Trainer, and the Bayesian Optimization (BO) module. Users will sequentially navigate through these components to identify and optimize RNA aptamers. Figure 1 provides an overview of the system’s architecture.

The VAE Trainer facilitates training of the pHMM-VAE model through an intuitive interface, allowing users to upload SELEX datasets in FASTA or FASTQ format. The system performs a series of computations, including the calculation of the most frequent sequence length in a single SELEX file and the automatic estimation of the fixed adapter sequences attached to the 5′ and 3′ ends. Additionally, minimum count thresholds can be configured to filter noisy data, contributing to a reduction in the overall cost of training. The training process supports concurrent execution on CPUs and CUDA devices using semaphore techniques, thereby enabling the training of multiple models with different seed values simultaneously. Users can select the best-performing model for further analysis. Furthermore, the training process can be paused, resumed, or deleted during training, allowing users to manage the progress of their experiments and enabling parameter adjustments and early feedback for time-consuming learning tasks.

The VAE Trainer can be visualized and manipulated through the VAE Viewer, which provides a user-friendly interface for exploring the latent space of the pHMM-VAE model via Plotly.js. This interface enables users to toggle between Gaussian Mixture Models (GMM) (Reynolds 2009) trained on the latent space, enabling the identification of clusters and motifs. Furthermore, the availability of features of encoding and decoding sequences enables direct manipulation of sequences within the latent space. Download options include both CSV and FASTA file formats. Additionally, the system supports downloads based on clusters, facilitating the extraction of sequences for further analysis. The viewer also supports motif output through WebLogo (Crooks *et al.* 2004) generation, highlighting motif regions.

In the GMM trainer module, RaptGen-UI trains multiple GMM models on the latent space and evaluate them utilizing Bayesian Information Criterion (BIC) (Schwarz 1978) values. The BIC is a criterion composed of two opposing factors: the likelihood of the data given the model, and a penalty term based on the number of parameters. A lower BIC value indicates that the model better fits the statistical distribution of the original data with appropriate number of parameters, avoiding over-fitting. RaptGen-UI selects the model with the lowest BIC value and utilize the mean points of it as the initial sequences for BO module mentioned later. By using BIC, users can acquire and measure a feasible number of sequences that appropriately and sufficiently cover all clusters in the latent space.

The BO module facilitates iterative optimization over the latent space of the pHMM-VAE. Initial values can be derived from GMM model averages or user-provided CSV files, and users can specify the number of sequences to query each iteration and the column to use as the objective function. The BO module uses BoTorch (Balandat *et al.* 2020), which applies SingleTaskGP by default. The system supports saving and resuming optimization sessions. This functionality enables users to utilize the system consistently during prolonged wet lab experiments and facilitates concurrent optimization processes, addressing the practical constraints of wet lab research while maximizing computational efficiency and experimental throughput.

## 4 Conclusion

This study presents RaptGen-UI, a software tool designed to streamline the identification and optimization of RNA aptamers. By integrating LSBO techniques with an intuitive user interface, the tool empowers researchers to efficiently conduct sequence analysis and motif identification while ensuring data security.

Future developments aim to expand the capabilities of this tool. While the current version focuses on RaptGen, to extend support to additional LSBO frameworks, such as RfamGen (Sumi *et al.* 2024), is under consideration. Furthermore, there are plans to implement multi-objective optimization in the BO module, thus broadening the scope of its applications.

The proposed software is designed for rapid deployment, making it suitable for addressing urgent therapeutic needs such as emerging infectious diseases. For instance, during the COVID-19 pandemic, various methods were employed to identify potential therapeutic agents against SARS-CoV-2. Among these methods, the use of RaptGen has identified the high-affinity RNA aptamer SPA1 for the SARS-CoV-2 spike protein (Adachi *et al.* 2024). This paper demonstrates the potential of RaptGen to facilitate a more extensive and scalable engagement in the identification of aptamers in the context of drug discovery. The RaptGen-UI is expected to be a valuable asset in RNA aptamer research.

## Data Availability

Source code and documentation are freely available at https://github.com/hmdlab/RaptGen-UI.
